# Clinical efficacy of anti-vascular endothelial growth factor versus panretinal photocoagulation for patients with proliferative diabetic retinopathy: A protocol for systematic review and meta-analysis

**DOI:** 10.1097/MD.0000000000025682

**Published:** 2021-04-30

**Authors:** Yuxian Lin, Xiaowei Zheng, QiuJie Chen, Ruibin Wu

**Affiliations:** Department of Ophthalmology, The First Affiliated Hospital of Shantou University Medical College, Guangdong, China.

**Keywords:** anti-vascular endothelial growth factor, meta, panretinal photocoagulation, proliferative diabetic retinopathy, protocol

## Abstract

**Background:**

The argument on the optimal treatment for patients with proliferative diabetic retinopathy (PDR) remains to be resolved. Therefore, the primary objective of the present study was to evaluate the clinical efficacy of anti-vascular endothelial growth factor (anti-VEGF) therapy versus panretinal photocoagulation (PRP) for patients with PDR.

**Methods:**

Two independent investigators followed The Preferred Reporting Items for Systematic Reviews and Meta-Analyses reporting guidelines and the recommendations of the Cochrane Collaboration to conduct this meta-analysis. The electronic databases of EMBASE, PubMed, Cochrane Library, and Web of Science were searched from the inception to April 2021 using the following key terms: “proliferative diabetic retinopathy,” “anti-vascular endothelial growth factor,” and “panretinal photocoagulation,” for all relevant studies. We identified literature that met the following inclusion criteria: patients with PDR; studies focusing on assessing anti-VEGF therapy and PRP; the following outcome measures must be shown: anatomical outcomes, including complete regression and recurrence of neovascularization, mean change in best corrected vision acuity from baseline to the end of follow-up period. The Cochrane risk of bias tool was used to evaluate the risk of bias of included randomized clinical trials by 2 independent reviewers.

**Results:**

This protocol will provide a reliable theoretical basis for the following research.

**Trial registration number:**

10.17605/OSF.IO/UHYDR.

## 1 Introduction

The prevalence of diabetes mellitus is currently increasing. Proliferative diabetic retinopathy (PDR) is the leading cause of blindness in working-age adults in the United States, with 12,000 to 24,000 new cases annually.^[1]^ It is characterized by the growth of neovascular vessels, which are prone to leakage, bleeding, and the development of vitreoretinal membranes and tractional retinal detachment. It can also invade the anterior segment and cause neovascular glaucoma or ischemia.^[2,3]^

Treatment options for PDR include vitrectomy, anti-vascular endothelial growth factor (anti-VEGF) therapy, and panretinal photocoagulation (PRP). Vitrectomy is typically performed in cases of opaque vitreous hemorrhage or tractive retinal detachment that threatens or involves the macula.^[4–6]^ PRP has been the mainstay of treatment for PDR. The proposed mechanisms of action include the reduction of ischemic drive through the ablation of the hypoxic retina and the increase of the partial pressure of oxygen in the vitreous cavity. Despite immediate treatment with PRP, a small number of patients develop serious complications, including tractive retinal detachment, diabetic vitreous hemorrhage, and neovascularglaucoma.^[7,8]^ Although PRP has been the standard of care for more than 40 years, recent clinical trials indicate that anti-VEGF therapy can replace PRP in the treatment of PDR for at least 2 years, depending on access to treatment and patient compliance.^[9,10]^

A systematic review in 2015 investigated the use of anti-VEGF therapy in PDR, but pooled analyses comparing anti-VEGF therapy to PRP included only 2 trials.^[11]^ Subsequently, new evidence from randomized clinical trials (RCTs) was published in the literature.^[1,10,12]^ Some important information may be obtained if these studies are analyzed. The argument on the optimal treatment for patients with PDR remains to be resolved. Therefore, the primary objective of the present study was to evaluate the clinical efficacy of anti-VEGF therapy versus PRP for patients with PDR.

## 2 Materials and methods

### 2.1 Search strategy

The systematic review protocol has been registered on Open Science Framework registries. Two independent investigators followed The Preferred Reporting Items for Systematic Reviews and Meta-Analyses reporting guidelines and the recommendations of the Cochrane Collaboration to conduct this meta-analysis. The electronic databases of EMBASE, PubMed, Cochrane Library, and Web of Science were searched from the inception to April 2021 using the following key terms: “proliferative diabetic retinopathy,” “anti-vascular endothelial growth factor,” and “panretinal photocoagulation,” for all relevant studies. Additionally, the reference lists from published original articles and relevant reviews were assessed to identify more relevant studies. Only English publications were included. Ethical approval was not necessary because the present meta-analysis was performed on the basis of previous published studies.

### 2.2 Inclusion and exclusion criteria

We identified literature that met the following inclusion criteria: patients with PDR; studies focusing on assessing anti-VEGF therapy and PRP; the following outcome measures must be shown: anatomical outcomes, including complete regression and recurrence of neovascularization, mean change in best corrected vision acuity from baseline to the end of follow-up period. The exclusion criteria were: no comparison of anti-VEGF therapy and PRP; non-RCTs, abstracts, case reports, letters, conference articles, repeated studies, biochemical trials, meta-analyses, and reviews were also eliminated; lack of useful data in outcomes mentioned above.

### 2.3 Data extraction

The method of data extraction followed the approach outlined by the Cochrane Handbook for Systematic Reviews of Interventions. Two independent authors extracted the following descriptive raw information from the selected studies: study characteristics such as author, publication year, study design; patient demographic details such as patients’ number, average age, body mass index, and gender ratio. The outcomes included anatomical outcomes, including complete regression and recurrence of neovascularization, mean change in best corrected vision acuity from baseline to the end of follow-up period. Where disagreement in the collection of data occurred, this was resolved through discussion. If the data were missing or could not be extracted directly, we contacted the corresponding authors to ensure that the information integrated. Otherwise, we calculated them with the guideline of Cochrane Handbook for Systematic Reviews of Interventions. If necessary, we would abandon the extraction of incomplete data.

### 2.4 Quality assessment

The Cochrane risk of bias tool was used to evaluate the risk of bias of included RCTs by 2 independent reviewers. The quality of RCTs was assessed by using following 7 items: random sequence generation, allocation concealment, blinding of participants and personnel, blinding of outcome assessment, incomplete outcome data, selective reporting, and other bias. Disagreement was resolved through discussion and consensus between the reviewers. Kappa values were used to measure the degree of agreement between the 2 reviewers and were rated as follows: fair, 0.40 to 0.59; good, 0.60 to 0.74; and excellent, 0.75 or more. Furthermore, we did not conduct publication bias because of the limited number of included studies.

### 2.5 Statistical analysis

Review Manager software (v 5.3; Cochrane Collaboration) was used for the meta-analysis. Extracted data were entered into Review Manager by the first independent author and checked by the second independent author. Odds ratio with a 95% confidence interval or mean difference with 95% CI were assessed for dichotomous outcomes or continuous outcomes, respectively. The heterogeneity was assessed by using the Q test and I^2^ statistic. An I^2^ value of <25% was chosen to represent low heterogeneity and an I^2^ value of >75% to indicate high heterogeneity. All outcomes were pooled on random-effect model. A *P* value of <.05 was considered to be statistically significant.

## 3 Discussion

We foresee several potential limitations with this systematic review: heterogeneity of clinical outcomes, substandard quality of existing studies, which are the focus of our project. Therefore, we will present our findings using descriptive methods, if necessary. Our hope is that the dissemination of this protocol will allow us to obtain feedback and constructive criticism of the methods before our study is conducted.

## Author contributions

Yuxian Lin and Xiaowei Zheng conceived, designed, and planed the study. Yuxian Lin, Xiaowei Zheng, and QiuJie Chen are recruiting the study participants and performing the interventions. Ruibin Wu supervised the study. Yuxian Lin, Xiaowei Zheng, and QiuJie Chen will interpret and analyze the data. Yuxian Lin drafted the manuscript. Ruibin Wu critically revised the manuscript for important intellectual content. All authors have full access to the manuscript and take responsibility for the study design. All authors have approved the manuscript and agree with submission.

**Conceptualization:** QiuJie Chen.

**Data curation:** Yuxian Lin, Xiaowei Zheng.

**Formal analysis:** Yuxian Lin, Xiaowei Zheng, QiuJie Chen.

**Funding acquisition:** Ruibin Wu.

**Investigation:** Yuxian Lin, Xiaowei Zheng.

**Methodology:** QiuJie Chen.

**Project administration:** Ruibin Wu.

**Resources:** Xiaowei Zheng.

**Software:** Yuxian Lin, QiuJie Chen.

**Supervision:** Ruibin Wu.

**Validation:** QiuJie Chen.

**Visualization:** Yuxian Lin, Xiaowei Zheng, QiuJie Chen.

**Writing – original draft:** Yuxian Lin.

**Writing – review & editing:** Ruibin Wu.
